# Diagnostic Accuracy of Adenosine Stress Cardiovascular Magnetic Resonance Following Acute ST-segment Elevation Myocardial Infarction Post Primary Angioplasty

**DOI:** 10.1186/1532-429X-13-62

**Published:** 2011-10-22

**Authors:** Dennis TL Wong, Michael CH Leung, Rajiv Das, Gary YH Liew, Kerry Williams, Benjamin K Dundon, Payman Molaee, Karen SL Teo, Ian T Meredith, Matthew I Worthley, Stephen G Worthley

**Affiliations:** 1 Cardiovascular Research Centre, Royal Adelaide Hospital & Department of Medicine, University of Adelaide, Adelaide, Australia; 2 Monash Cardiovascular Research Centre, Department of Medicine (MMC), MonashUniversity, and MonashHeart, Melbourne, Australia

## Abstract

**Background:**

Adenosine stress cardiovascular magnetic resonance (CMR) has been proven an effective tool in detection of reversible ischemia. Limited evidence is available regarding its accuracy in the setting of acute coronary syndromes, particularly in evaluating the significance of non-culprit vessel ischaemia. Adenosine stress CMR and recent advances in semi-quantitative image analysis may prove effective in this area. We sought to determine the diagnostic accuracy of semi-quantitative versus visual assessment of adenosine stress CMR in detecting ischemia in non-culprit territory vessels early after primary percutaneous coronary intervention (PCI) for ST-segment elevation myocardial infarction (STEMI).

**Methods:**

Patients were prospectively enrolled in a CMR imaging protocol with rest and adenosine stress perfusion, viability and cardiac functional assessment 3 days after successful primary-PCI for STEMI. Three short axis slices each divided into 6 segments on first pass adenosine perfusion were visually and semi-quantitatively analysed. Diagnostic accuracy of both methods was compared with non-culprit territory vessels utilising quantitative coronary angiography (QCA) with significant stenosis defined as ≥70%.

**Results:**

Fifty patients (age 59 ± 12 years) admitted with STEMI were evaluated. All subjects tolerated the adenosine stress CMR imaging protocol with no significant complications. The cohort consisted of 41% anterior and 59% non anterior infarctions. There were a total of 100 non-culprit territory vessels, identified on QCA. The diagnostic accuracy of semi-quantitative analysis was 96% with sensitivity of 99%, specificity of 67%, positive predictive value (PPV) of 97% and negative predictive value (NPV) of 86%. Visual analysis had a diagnostic accuracy of 93% with sensitivity of 96%, specificity of 50%, PPV of 97% and NPV of 43%.

**Conclusion:**

Adenosine stress CMR allows accurate detection of non-culprit territory stenosis in patients successfully treated with primary-PCI post STEMI. Semi-quantitative analysis may be required for improved accuracy. Larger studies are however required to demonstrate that early detection of non-culprit vessel ischemia in the post STEMI setting provides a meaningful test to guide clinical decision making and ultimately improved patient outcomes.

## Background

Vasodilator induced myocardial perfusion defects are widely used in both nuclear and magnetic resonance based non-invasive imaging studies to detect myocardial ischemia. It offers functional relevance not provided by angiographic assessment. Clinical routine measurements of myocardial perfusion can be performed effectively with single-photon emission computer tomography (SPECT) and positron emission tomography (PET) studies. Cardiovascular magnetic resonance (CMR) however provides superior spatial resolution with the ability to detect subendocardial defects [1-3] as well as additional benefits regarding the evaluation of valvular disease and excellent assessment of left ventricular structure, function and viability.

It is now well established that up to 20-30% of patients following an admission for an acute coronary syndrome will have a further cardiovascular event [4-6]. It has been recently shown that half of these events will be at a non-culprit site [4]. This is particularly an issue in the ST-segment elevation myocardial infarction (STEMI) setting where up to 40% 'significant' non-culprit angiographic disease is seen at primary percutaneous coronary intervention (PCI). While it is well established that intervening on a non-culprit lesion at the time of primary-PCI is associated with adverse outcomes [7-9] identifying an early non-invasive imaging modality to effectively identify non-culprit vessel ischemia, may identify high-risk lesions.

As there is uncertainty regarding the effectiveness of adenosine in the immediate post infarct period due to potential microvascular dysfunction in the infarcted territory, the focus of our study was to compare the effectiveness of semi- quantitative versus visual evaluation of adenosine stress CMR in detecting non-culprit ischemia in the post primary-PCI setting, compared to quantitative coronary angiography (QCA).

## Methods

### Study population

All subjects gave written informed consent in accordance with local human research and ethics committee approval. We prospectively studied patients with acute STEMI who underwent primary PCI, between April 2008 and April 2009. We defined STEMI as chest pain for at least 30 minutes and an ECG demonstrating ST-segment elevation of > 0.1 mV in ≥2 contiguous leads. Patients aged < 18 years, previous myocardial infarction in the same territory, atrioventricular block of grade II or higher, severe asthma of chronic obstructive airways disease, contraindications to CMR, (eg, pacemaker implantation or claustrophobia) contraindication to gadopentetate dimeglumine.(eg, known hypersensitivity to gadopentetate dimeglumine or creatinine clearance ≤60 ml/min/1.73 m^2^) or pregnancy were excluded from the study. All patients were advised not to drink tea or coffee within 24 hours before the examinations. All participants gave written consent to the study protocol. The adenosine stress CMR was performed on day 3 following primary PCI with non-culprit territories defined by quantitative coronary angiography data acquired at the initial primary PCI.

### Adenosine infusion protocol

Adenosine (Adenoscan^®^, Sanofi-Synthelabo) was infused at 140 μg/kg/min through an antecubital vein using an accurate syringe pump (Graseby^® ^3500). The target time of the infusion was 3 minutes, however if patients developed persistent or symptomatic 3^rd ^AV block, severe hypotension (systolic blood pressure < 90 mmHg) or bronchospasm, infusion was discontinued. The attending physicians had aminophylline for adenosine receptor antagonism, nitroglycerine for persistent chest pain, atropine for persistent AV block and a fully equipped crash trolley with defibrillator if required.

### CMR

All CMR studies were performed using a 1.5 T MRI scanner (Magnetom Avanto, Siemens, Germany) equipped with a dedicated cardiac software package and a cardiac phased array surface coil. During the last minute of adenosine infusion a gadolinium-based contrast agent (Dimeglumine gadopentetate, Magnevist, Bayer) was administered intravenously at 0.1 mmol/kg body weight (injection rate, 7 ml/s), followed by at least 30 mL saline flush at the same rate [10,11]. Perfusion imaging (echo time 1.08 ms, repetition time 2.2 ms, saturation recovery time 100 ms, shot time 100 ms, voxel size 2.5 × 1.9 × 10 mm; flip angle 10°) was performed every cardiac cycle during the first pass, using a T1-weighted fast low-angle single shot gradient-echo sequence (GRE). Parallel acquisition method using generalised autocalibrating partially parallel acquisition (GRAPPA) was utilised [12]. Three short axis slices, positioned from base to the apex of the left ventricle, were obtained. The same imaging sequence was repeated 20 minutes later without adenosine to obtain perfusion images at rest.

### CMR Analysis

#### Left ventricular function and late gadolinium enhancement analysis

Left ventricular ejection fraction (LVEF), volume and mass were measured upon cine images using commercially available software (MASS, Medis, The Netherlands). Papillary muscles and pericardial fat were excluded from calculations. In brief, the end-diastolic and end-systolic cine frames were identified for each slice and the endocardial and epicardial borders were manually traced. The end-diastolic and end-systolic volumes were then calculated using Simpson's true disk summation technique (i.e. sum of cavity sizes across all continuous slices), as previously described [13]. Late gadolinium enhancement (LGE) images were assessed both for scar mass and microvascular obstruction (MVO). Scar mass was semi-quantitatively quantified using the full width at half maximum technique (MASS, Medis, The Netherlands) while MVO was manually planimetered [14]. Microvascular obstruction on LGE imaging was defined as late hypo-enhancement within a hyper-enhanced region [15,16]. Microvascular obstruction was included in the calculation of the total scar mass.

#### Visual Analysis

**Figure 1:**
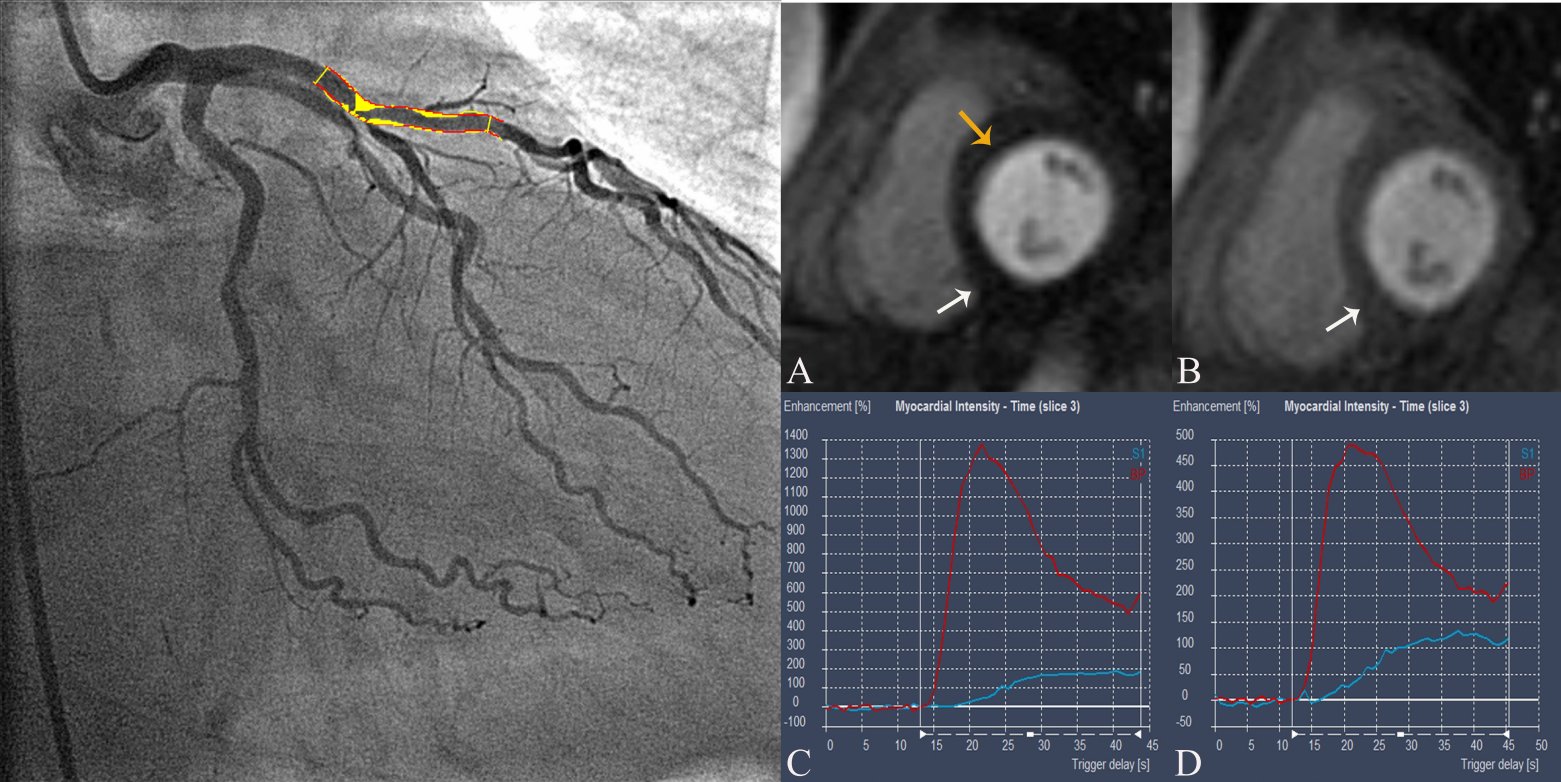
**Example of perfusion measurement in a patient with MPRI of 0.6 in the left anterior descending artery which has 81% stenosis on QCA**. A) Perfusion defect (yellow arrow) in anteroseptal wall on left ventricular short axis. Perfusion defect (white arrow) in inferior wall most likely secondary to microvascular obstruction within infarct B) No perfusion defect at rest in anteroseptal wall of left ventricle. Perfusion defect (white arrow) in inferior wall within infarct most likely secondary to microvascular obstruction C) Signal intensity-time curve in anteroseptal wall at stress. Red line represents signal intensity-time curve for left ventricle while blue line represents signal intensity-time curve for anteroseptum segment D) Signal intensity-time curve in anteroseptal wall at rest. Red line represents signal intensity-time curve for left ventricle while blue line represents signal intensity-time curve for anteroseptum segment.

**Figure 2:**
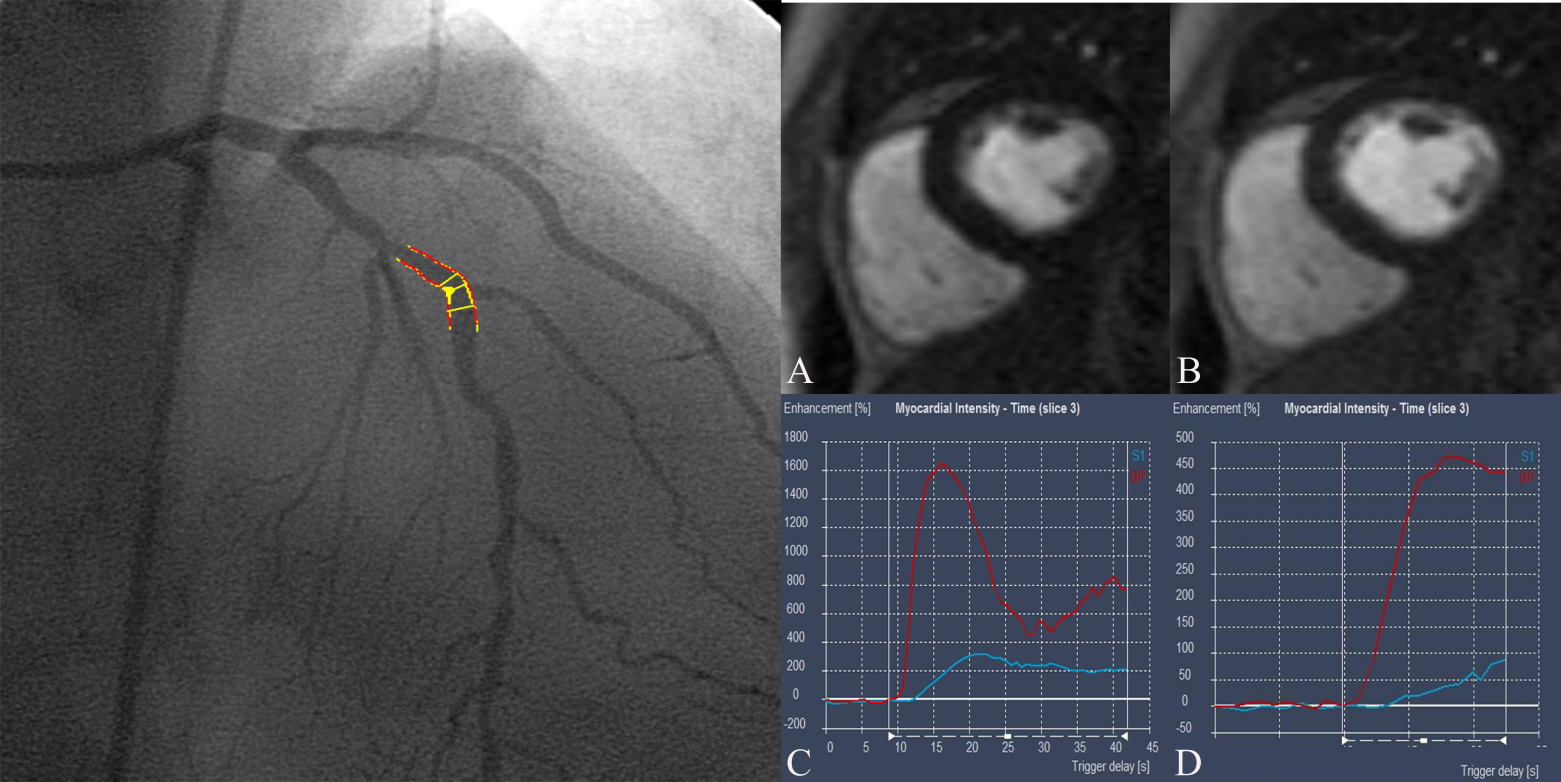
**Example of myocardial perfusion measurement in a patient with MPRI of 1.96 in the left anterior descending artery which has 43% stenosis on QCA**. A) No evidence of perfusion defect in the left anterior descending artery territory at stress B) No evidence of perfusion defect in the left anterior descending artery territory at rest C) Signal intensity-time curve of anteroseptal wall of left ventricle at stress D) Signal intensity-time curve of anteroseptal wall of left ventricle at rest.

Visual analysis of the CMR perfusion was done off-line by consensus of two experienced observers by an examiner blinded to coronary angiography findings. Rest and stress perfusion and the late gadolinium enhancement images of three short axis sections (base, mid and apex) were viewed side by side. If the signal intensity on stress perfusion appeared lower in an area of myocardium for at least three dynamic images compared with remote myocardium, it was considered to be ischemic, as previous described [17]. If the same signal intensity abnormality was seen in the rest and stress perfusion images and there was no evidence of scar on late contrast enhanced images, the defect was considered an artefact [17]. (Figure 1 &2)

#### Semi-quantitative analysis

The endocardial and epicardial contours of three short axis sections (base, mid and apex) were traced (QMASS, Version 7.2, Medical Imaging Solutions, Leiden, the Netherlands) and corrected manually for displacements (eg. breathing) by an examiner blinded to coronary angiography findings.

**Figure 3:**
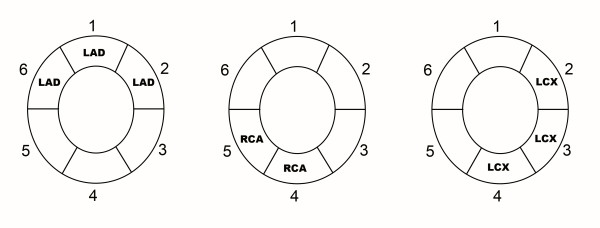
**Diagrams show segments assigned to vascular regions**. On every section, segments 6, 1, and 2 were assigned to the left anterior descending artery *(LAD); *segments 2, 3, and 4, to the circumflex artery *(LCX); *and segments 4 and 5, to the right coronary artery *(RCA)*.

All three short axis sections were divided into six equiangular segments starting in a clockwise direction from the anterior septal insertion of the right ventricle (Figure 3) [18]. Segments were assigned to vascular regions according to the segmental model of the American Society of Echocardiography, with modifications to correct for variable coronary dominances as previously described [19]. Segments 6, 1 and 2 were assigned to the left anterior descending artery; segments 2, 3 and 4 were assigned to the circumflex artery; and segments 4 and 5 were assigned to the right coronary artery.

Within each segment, signal intensity was measured by defining regions of interest that excluded the inner 10% and outer 30% of the myocardium to get stronger weighting of the subendocardium and reduce influences from the LV as previously described [3] using commercially available software (QMASS Version 7.2, Medis, Netherlands).

The subendocardial signal intensity-time curves were generated for all segments by obtaining signal intensity on consecutive images before and during arrival of contrast material. The signal intensity-time curve for the left ventricle was generated on the basal section as a measure of input function. (Figure 1 &2)The maximal initial upslope of every signal intensity-time curve was determined by using a sliding window with a four- point linear fit for the myocardium and a three-point fit for the left ventricle as previously described [19]. Myocardial upslopes were then divided by left ventricular upslopes to correct them for the timing and dispersion of the bolus contrast material.

For each segment, the MPRI was calculated by dividing the value at stress over rest. MPRI of a vascular region was determined by analysis of only the second smallest value of MPRI in the vascular region with the use of a cutoff of 1.1 [3].

### Quantitative Coronary Angiography

The quantitative coronary analyses of the non-culprit stenotic lesions were performed on a dedicated software tool (QCA-CMS 6.0, Medis, Netherlands). Orthogonal views of coronary angiograms were obtained during primary-PCI for analysis. Intracoronary gliceryl trinitrate (GTN) was routinely used prior to assessment of non-culprit vessel angiographic analysis. The quantitative measurements were performed on end-diastolic frames of the angiograms by an investigator who was blinded to the results of the adenosine stress CMR results. The reference diameter, lesion length and minimal luminal diameter were measured and the reference diameter was determined by software at the interpolation line between the normal segments proximal and distal to the stenosis [20,21]. The diameter stenosis (DS) was calculated as percent stenosis of the reference diameter and we defined a significant stenosis as ≥70%.

### Statistical Analysis

Continuous variables were expressed as means ± standard deviations. The diagnostic accuracy in terms of sensitivity, specificity and positive and negative predictive values of CMR for detecting a significant coronary stenosis (≥70%) on QCA was determined. Comparisons between ordinal variables were made using Chi square analysis. A probability (p) value < 0.05 was considered significant. Receiver operating characteristic (ROC) analyses was performed to evaluate the diagnostic potential of MPRI derived by semi-quantitative analysis in detecting non-culprit territory stenosis. Area under the curve (AUC) was determined.

## Results

A total of 61 patients were recruited to undergo contrast enhanced CMR 3 ± 2 days post STEMI. Five patients had history of asthma, three patients were claustrophobic and unable to complete the CMR study and three patients had poor quality images due to rapid atrial fibrillation. A total of 50 patients successfully underwent adenosine stress CMR. Baseline demographics are shown in Table 1. One hundred non-culprit vessels were identified by QCA, 31 left anterior descending artery, 43 left circumflex artery and 26 right coronary artery lesions. There were 7 (14%) patients with non- culprit territory stenosis. Mean left ventricular ejection fraction of patients was 57 ± 11% (mean ± SD).

**Table 1:** Patient demographics

Demographic	
**Age, mean ± SD**	59 ± 12
**Male: Female**	43: 7
**Risk factor, n (%)**	
**Current smoker**	16 (32)
**Diabetes mellitus**	8 (16)
**Hypertension**	20 (40)
**Hypercholesterolemia**	16 (32)
**Previous MI**	4 (8)
**Family history of IHD**	12 (24)
**Stroke**	3 (6)
**Peripheral vascular disease**	4 (8)
**Medication on discharge, n (%)**	
**Aspirin**	50 (100)
**Clopidogrel**	50 (100)
**β-blocker**	45 (90)
**Statin**	49 (98)
**ACE-inhibitor**	48 (95)
**Infarct location, n (%)**	
**Anterior**	21 (41)
**Non-anterior**	29 (59)

All 50 patients completed the adenosine stress CMR study, which was undertaken at a mean of 3 ± 2 days post primary PCI. During adenosine stress CMR perfusion, most patients reported at least one mild symptoms of flushing, breathlessness or chest discomfort. No patients developed significant AV block or hypotension. The heart rate and rate-pressure product obtained at stress were significantly higher than those obtained at rest. (Table 2)

**Table 2:** Summary of Hemodynamic Data

Parameter	Adenosine CMR Imaging Measurements
**Heart rate (beats/min)**	
Rest	64 ± 9
Stress	78 ± 12
**Systolic blood pressure (mm Hg)**	
Rest	137 ± 25
Stress	140 ± 24
**Rate-pressure product**	
Rest	8809 ± 2334
Stress	11008 ± 2873

Data are means ± standard deviations.

Mean minimal luminal diameter of the stenotic lesions was 0.9 ± 0.6 mm with a mean reference diameter of 3.3 ± 0.9 mm, yielding a mean diameter stenosis of 79 ± 4.4%. Mean length of the stenotic lesions was 16.5 ± 5.3 mm. The MPRI in coronary arteries with diameter stenosis < 70% was higher (2.18 ± 0.72, mean ± SD) than MPRI in coronary arteries with diameter stenosis ≥70% (0.98 ± 0.48, mean ± SD).

**Figure 4:**
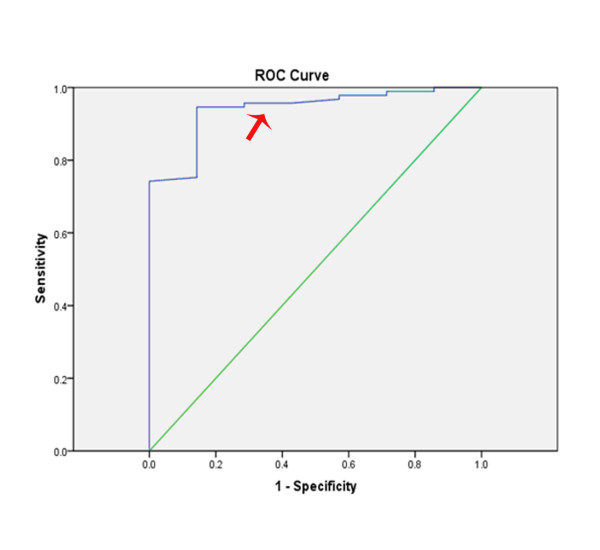
**Receiver operator curve of MPRI in detection of non-culprit territory stenosis ≥ 70%**. The area under the curve for MPRI was 0.94. Red arrow indicates the MPRI cut-off of 1.1.

Visual assessment of the adenosine stress CMR study compared to 'gold standard' QCA showed 96% sensitivity, 50% specificity, 97% positive predictive value, 43% negative predictive value and diagnostic odds ratio of 22.5. The semi-quantitative assessment of the adenosine stress CMR study utilising a MPRI cut- off of 1.1 compared to 'gold standard' QCA showed 99% sensitivity, 67% specificity, 97% positive predictive value, 86% negative predictive value and diagnostic odds ratio of 180. On ROC analysis, the area under the curve (AUC) of MPRI in detecting non-culprit territory stenosis ≥70% was 0.94 (Figure 4). A retrospectively determined cut-off value that maximised the sensitivity and specificity of MPRI in our study was also determined. Utilising a MPRI cut-off of 1.15, the sensitivity was 86%, 95% specificity, 55% positive predictive value and 99% negative predictive value and diagnostic odds ratio of 107.

### Semi-quantitative analysis reproducibility

Semi-quantitative analysis showed good reproducibility between observers in 10 randomly selected patients. The intraclass coefficient was 0.84 (p < 0.001).

## Discussion

Our study shows that semi-quantitative assessment of adenosine stress CMR has good diagnostic accuracy in detecting non-culprit artery stenosis early after primary-PCI for STEMI, with a twofold improvement in negative predictive value compared to visual assessment.

Assessment of non-culprit artery stenosis after primary-PCI is essential as up to 40% of patients presenting with STEMI have multivessel disease [22]. The current clinical recommendation is that a deferred angioplasty strategy of non-culprit lesions should remain the standard interventional approach in patients with STEMI undergoing primary-PCI. It is accepted that intervening on non-culprit vessels at the time of primary-PCI is associated with adverse outcomes [8,9]. A recent meta-analysis that included 18 studies and more than 40 000 patients showed that multivessel PCI was associated with the highest rates of death in short and long-term follow up when compared with culprit-lesion PCI and staged PCI [7].

However it has been recently shown that half of all cardiovascular events that occur following an acute coronary syndrome occur at a non-culprit lesion [4]. While the PROSPECT study showed in 697 patients that intravascular ultrasound derived independent risk factors that would indicate a non-culprit lesion was associated with a recurrent clinical event included plaque burden and thin-cap fibroatheroma on radiofrequency analysis, no assessment of ischemia was performed. As many events following ACS occur without warning symptoms, an early non-invasive reliable test may indicate high risk non-culprit lesions that ultimately may be proven to be risky to adopt a 'symptom' driven strategy for revascularisation.

Myocardial perfusion assessment can be performed visually by comparing the rest and stress scans together on a viewing platform. This allows recognition of perfusion defects and discrimination from artefacts [23]. The sensitivity utilising the visual analysis method ranged from 81 to 91% in previous studies while specificity ranged from 62 to 85% [24-26]. The sensitivity of visual analysis in our study (97%) was comparable to previous studies. However, our specificity of 50% was lower than previous published studies. This could be explained by the high prevalence of diabetes (16%), hypertension (40%) and hypercholesterolemia (32%) in our patients causing microcirculatory dysfunction. In addition, the presence of acutely injured myocardium or variable amounts of scar may have an unpredictable effect on the visual interpretation of myocardial perfusion. The presence of microvascular obstruction in the infarct region could also contribute to the difficulty in assessing for dynamic change visually in remote myocardium. There is also no current consensus in the definition of an artefact, most commonly dark rim artefact, based on the persistence of a defect. The use of an arbitrary cut off such as persistence of less than 3 phases compared to 6 phases described by other groups for artefact may explain the low specificity. The negative predictive value of (47%) was suboptimal with the visual assessment methodology for evaluating ischemia.

Semi-quantitative assessment of myocardial perfusion had also been previously studied and validated with other techniques such as fractional flow reserve (FFR) and quantitative coronary angiography (QCA). However different methodologies had yielded different cut-off values for functional significant ischemia [3,18,19]. These studies examined the diagnostic accuracy of semi-quantitative assessment for detecting significant coronary artery disease in patients referred for investigation of suspected angina. The sensitivity of the subendocardial methodology described by Barmeyer et al using a cutoff MPRI value of 1.21 was 84% while the specificity was 75% [18]. On the other hand the sensitivity of the subendocardial methodology described by Nagel et al using a cutoff MPRI value of 1.1 was 88% with a specificity of 90% [3]. Nonetheless, these cutoff values were retrospectively determined by ROC analysis which may lead to an optimistic accuracy of the technique.

To our knowledge, no studies had ever examined the diagnostic accuracy of semi-quantitative assessment of adenosine stress CMR in patients post primary-PCI for STEMI. Hence, no previous methodologies or cut-off values had ever been described in this setting. We chose the methodology previously validated by Nagel et al and prospectively examined the diagnostic accuracy of this method compared to QCA. In addition, our retrospectively determined MPRI of 1.15 was similar to the cut-off value of 1.1 validated by Nagel et al. This showed excellent sensitivity, positive predictive value and negative predictive value. The small number of patients with severe non-culprit vessel stenosis could have contributed to the diagnostic accuracy. Therefore further larger studies with higher prevalence of non-culprit stenosis are required to compare the diagnostic accuracy of semi-quantitative and visual analysis of adenosine stress CMR post STEMI.

## Limitations

A relatively small size of the patient population might represent a limitation of the study. A larger sample size would have allowed subgroup analysis of the influence of infarct size and microvascular obstruction on MPRI in non-culprit territory.

Despite the excellent results of adenosine stress CMR for accurate detection of non- culprit territory stenosis, there are some important limitations. Claustrophobia remains problematic while adenosine is contraindicated in asthmatics and patients with high degree AV block. Patients with permanent pacemakers would also be ineligible for this diagnostic test.

Although we performed both visual and semi-quantitative analysis, the CMR protocol was optimised for visual analysis. For semi-quantitative analysis, generating the signal intensity time curves for calculation of the upslope from the CMR images although reproducible is time consuming. Manual segmentation of the myocardium and correction for diaphragmatic motion had to be performed. In addition, although the MPRI cut-off derived from our study approximates the MPRI cut-off of 1.1 previously described, generalising this cut-off across different CMR protocols will require further studies. This therefore reduces the use of this technique for clinical routine measurements and hence majority of centers continue to use visual analysis for the assessment of clinical myocardial perfusion scans.

## Conclusions

Adenosine stress CMR allows accurate detection of non-culprit territory stenosis in patients successfully treated with primary percutaneous intervention post STEMI. Semi-quantitative analysis may be required for improved accuracy. Larger studies are however required to demonstrate that early detection of non-culprit vessel ischemia in the post STEMI setting provides a meaningful test to guide clinical decision making and ultimately improved patient outcomes.

## Abbreviations list

CMR: Cardiovascular Magnetic Resonance; STEMI: ST-segment Elevation Myocardial Infarction; PCI: Percutaneous Coronary Intervention; QCA: Quantitative Coronary Angiography; MPRI: Myocardial Perfusion Reserve Index; LGE: Late gadolinium enhancement.

## Competing interests

The authors declare that they have no competing interests.

## Authors' contributions

DW conceived the study, participated in its design and coordination, acquired, analysed and interpreted the data, drafted the manuscript. ML conceived the study, participated in its design and coordination, acquired the data and helped draft the manuscript. RD helped acquire the data and the drafting of the manuscript. GL helped acquire and analyse the data, helped to draft the manuscript. KW helped acquire the data. BD helped acquire the data and helped to draft the manuscript. PM helped acquire the data and helped draft the manuscript. KT conceived the study, participated in its design and coordination, helped to draft the manuscript. IM conceived of the study and helped to draft the manuscript. MW conceived of the study and helped to draft the manuscript. SW conceived of the study, participated in its design and coordination and helped to draft the manuscript.

All authors read and approved the final manuscript.

## References

[B1] Atkinson DJ Burstein D Edelman RR First-pass cardiac perfusion: evaluation with ultrafast MR imaging Radiology 1990 174 757 762 2305058 10.1148/radiology.174.3.2305058

[B2] Cullen JH Horsfield MA Reek CR Cherryman GR Barnett DB Samani NJ A myocardial perfusion reserve index in humans using first-pass contrast-enhanced magnetic resonance imaging Journal of the American College of Cardiology 1999 33 1386 1394 10.1016/S0735-1097(99)00004-2 10193743

[B3] Nagel E Klein C Paetsch I Hettwer S Schnackenburg B Wegscheider K Fleck E Magnetic resonance perfusion measurements for the noninvasive detection of coronary artery disease Circulation 2003 108 432 437 10.1161/01.CIR.0000080915.35024.A9 12860910

[B4] Stone GW Maehara A Lansky AJ de Bruyne B Cristea E Mintz GS Mehran R McPherson J Farhat N Marso SP A prospective natural-history study of coronary atherosclerosis N Engl J Med 2011 364 226 235 10.1056/NEJMoa1002358 21247313

[B5] Zapata GO Lasave LI Kozak F Damonte A Meirino A Rossi M Carbo S Pollice A Paolasso E Picabea E Culprit-only or multivessel percutaneous coronary stenting in patients with non-ST-segment elevation acute coronary syndromes: one-year follow-up J Interv Cardiol 2009 22 329 335 10.1111/j.1540-8183.2009.00477.x 19515083

[B6] Brener SJ Murphy SA Gibson CM DiBattiste PM Demopoulos LA Cannon CP Efficacy and safety of multivessel percutaneous revascularization and tirofiban therapy in patients with acute coronary syndromes Am J Cardiol 2002 90 631 633 10.1016/S0002-9149(02)02569-9 12231091

[B7] Vlaar PJ Mahmoud KD Holmes DR Jr van Valkenhoef G Hillege HL van der Horst IC Zijlstra F de Smet BJ Culprit Vessel Only Versus Multivessel and Staged Percutaneous Coronary Intervention for Multivessel Disease in Patients Presenting With ST-Segment Elevation Myocardial Infarction A Pairwise and Network Meta-Analysis J Am Coll Cardiol 2011 58 692 703 10.1016/j.jacc.2011.03.046 21816304

[B8] Kornowski R Mehran R Dangas G Nikolsky E Assali A Claessen BE Gersh BJ Wong SC Witzenbichler B Guagliumi G Prognostic Impact of Staged Versus "One-Time" Multivessel Percutaneous Intervention in Acute Myocardial Infarction Analysis From the HORIZONS-AMI (Harmonizing Outcomes With Revascularization and Stents in Acute Myocardial Infarction) Trial J Am Coll Cardiol 2011 58 704 711 10.1016/j.jacc.2011.02.071 21816305

[B9] Toma M Buller CE Westerhout CM Fu Y O'Neill WW Holmes DR Jr Hamm CW Granger CB Armstrong PW Non-culprit coronary artery percutaneous coronary intervention during acute ST-segment elevation myocardial infarction: insights from the APEX-AMI trial Eur Heart J 2010 31 1701 1707 10.1093/eurheartj/ehq129 20530505

[B10] Lyne JC Gatehouse PD Assomull RG Smith GC Kellman P Firmin DN Pennell DJ Direct comparison of myocardial perfusion cardiovascular magnetic resonance sequences with parallel acquisition J Magn Reson Imaging 2007 26 1444 1451 10.1002/jmri.21167 17968886

[B11] Kramer CM Barkhausen J Flamm SD Kim RJ Nagel E Standardized cardiovascular magnetic resonance imaging (CMR) protocols, society for cardiovascular magnetic resonance: board of trustees task force on standardized protocols J Cardiovasc Magn Reson 2008 10 35 10.1186/1532-429X-10-35 18605997 PMC2467420

[B12] Griswold MA Jakob PM Heidemann RM Nittka M Jellus V Wang J Kiefer B Haase A Generalized autocalibrating partially parallel acquisitions (GRAPPA) Magn Reson Med 2002 47 1202 1210 10.1002/mrm.10171 12111967

[B13] Teo KS Carbone A Piantadosi C Chew DP Hammett CJ Brown MA Worthley SG Cardiac MRI assessment of left and right ventricular parameters in healthy Australian normal volunteers Heart Lung Circ 2008 17 313 317 10.1016/j.hlc.2007.11.136 18314390

[B14] Amado LC Gerber BL Gupta SN Rettmann DW Szarf G Schock R Nasir K Kraitchman DL Lima JA Accurate and objective infarct sizing by contrast-enhanced magnetic resonance imaging in a canine myocardial infarction model J Am Coll Cardiol 2004 44 2383 2389 10.1016/j.jacc.2004.09.020 15607402

[B15] Hombach V Grebe O Merkle N Waldenmaier S Hoher M Kochs M Wohrle J Kestler HA Sequelae of acute myocardial infarction regarding cardiac structure and function and their prognostic significance as assessed by magnetic resonance imaging Eur Heart J 2005 26 549 557 15713695 10.1093/eurheartj/ehi147

[B16] Nijveldt R Beek AM Hofman MB Umans VA Algra PR Spreeuwenberg MD Visser CA van Rossum AC Late gadolinium-enhanced cardiovascular magnetic resonance evaluation of infarct size and microvascular obstruction in optimally treated patients after acute myocardial infarction J Cardiovasc Magn Reson 2007 9 765 770 10.1080/10976640701545008 17891613

[B17] Ingkanisorn WP Kwong RY Bohme NS Geller NL Rhoads KL Dyke CK Paterson DI Syed MA Aletras AH Arai AE Prognosis of negative adenosine stress magnetic resonance in patients presenting to an emergency department with chest pain Journal of the American College of Cardiology 2006 47 1427 1432 10.1016/j.jacc.2005.11.059 16580532

[B18] Barmeyer AA Stork A Muellerleile K Tiburtius C Schofer AK Heitzer TA Hofmann T Adam G Meinertz T Lund GK Contrast-enhanced cardiac MR imaging in the detection of reduced coronary flow velocity reserve Radiology 2007 243 377 385 10.1148/radiol.2432060331 17456867

[B19] Al-Saadi N Nagel E Gross M Bornstedt A Schnackenburg B Klein C Klimek W Oswald H Fleck E Noninvasive detection of myocardial ischemia from perfusion reserve based on cardiovascular magnetic resonance Circulation 2000 101 1379 1383 10736280 10.1161/01.cir.101.12.1379

[B20] de Feyter PJ Serruys PW Davies MJ Richardson P Lubsen J Oliver MF Quantitative coronary angiography to measure progression and regression of coronary atherosclerosis. Value, limitations, and implications for clinical trials Circulation 1991 84 412 423 2060112 10.1161/01.cir.84.1.412

[B21] Garrone P Biondi-Zoccai G Salvetti I Sina N Sheiban I Stella PR Agostoni P Quantitative coronary angiography in the current era: principles and applications J Interv Cardiol 2009 22 527 536 10.1111/j.1540-8183.2009.00491.x 19627430

[B22] Corpus RA House JA Marso SP Grantham JA Huber KC Jr Laster SB Johnson WL Daniels WC Barth CW Giorgi LV Rutherford BD Multivessel percutaneous coronary intervention in patients with multivessel disease and acute myocardial infarction Am Heart J 2004 148 493 500 10.1016/j.ahj.2004.03.051 15389238

[B23] Di Bella EV Parker DL Sinusas AJ On the dark rim artifact in dynamic contrast-enhanced MRI myocardial perfusion studies Magn Reson Med 2005 54 1295 1299 10.1002/mrm.20666 16200553 PMC2377407

[B24] Paetsch I Jahnke C Wahl A Gebker R Neuss M Fleck E Nagel E Comparison of dobutamine stress magnetic resonance, adenosine stress magnetic resonance, and adenosine stress magnetic resonance perfusion Circulation 2004 110 835 842 10.1161/01.CIR.0000138927.00357.FB 15289384

[B25] Sakuma H Suzawa N Ichikawa Y Makino K Hirano T Kitagawa K Takeda K Diagnostic accuracy of stress first-pass contrast-enhanced myocardial perfusion MRI compared with stress myocardial perfusion scintigraphy Ajr 2005 185 95 102 15972407 10.2214/ajr.185.1.01850095

[B26] Ishida N Sakuma H Motoyasu M Okinaka T Isaka N Nakano T Takeda K Noninfarcted myocardium: correlation between dynamic first-pass contrast-enhanced myocardial MR imaging and quantitative coronary angiography Radiology 2003 229 209 216 10.1148/radiol.2291021118 12944596

